# Clinical and Radiological Profile of Extrapulmonary Tuberculosis in Elderly People Attending a Tertiary Care Hospital: A Prospective Cohort Study

**DOI:** 10.7759/cureus.91779

**Published:** 2025-09-07

**Authors:** Arun Prakaash, Giridhar Murali, Priyamalini D, Elangovan Raman, Chandra Mouli, Vikram V J

**Affiliations:** 1 Department of Geriatric Medicine, Madras Medical College and Rajiv Gandhi Government General Hospital, Chennai, IND; 2 Department of Critical Care Medicine, Christian Medical College, Vellore, IND; 3 Department of Internal Medicine, Madras Medical College and Rajiv Gandhi Government General Hospital, Chennai, IND; 4 Upgraded Institute of Otorhinolaryngology, Madras Medical College and Rajiv Gandhi Government General Hospital, Chennai, IND

**Keywords:** elderly population, extrapulmonary tuberculosis, immunocompromised individuals, lymph nodes, tb mortality

## Abstract

Extrapulmonary tuberculosis (EPTB) poses distinct diagnostic and therapeutic challenges in the elderly due to subtle clinical signs, weakened immunity, and multiple coexisting health conditions. This observational study, conducted over 15 months at a tertiary care hospital in Chennai, India, included 120 patients aged 60 and above diagnosed with EPTB. The research examined clinical features, laboratory findings, comorbidities, and treatment history to identify patterns and risk factors. Disseminated tuberculosis (TB) was the most frequent form, with common symptoms including altered mental status and reduced functional capacity. The findings underscore the importance of age-adapted diagnostic approaches and comprehensive care strategies to enhance outcomes in older adults. In this study, the most frequently observed laboratory abnormalities among elderly patients with EPTB were hyponatremia, hypoalbuminemia, and anemia. This elevated occurrence suggests a need for further investigation through larger studies to determine the underlying cause. Comorbidities were present in 71.6% of cases, with diabetes mellitus being the most commonly seen in 56.7% of patients. Furthermore, 31.6% had multiple coexisting conditions, most frequently a combination of diabetes and hypertension (22.5%). These comorbidities may influence disease progression and complicate treatment response, underscoring the need for routine screening and appropriate management of associated health conditions in all TB patients.

## Introduction

Extrapulmonary tuberculosis (EPTB) refers to tuberculosis (TB) infections that occur outside the pulmonary system, affecting various organs, including the lymph nodes, pleura, bones, joints, central nervous system (CNS), genitourinary tract, abdomen, skin, and eyes [1]. It represents a significant portion of total TB cases, particularly among immunocompromised individuals, including the elderly and those with HIV. The disease typically spreads through hematogenous or lymphatic routes from a primary pulmonary site or may result from the reactivation of latent infection. Diagnosing EPTB poses challenges due to its diverse clinical presentations and the difficulty in accessing affected tissues. Traditional diagnostic methods, such as smear microscopy, are often insufficient due to the low bacterial load, making histopathology, advanced imaging, and molecular techniques essential for accurate identification.

Radiological investigations play a pivotal role in the diagnosis of EPTB, particularly in elderly patients, where clinical presentations are often non-specific. Imaging modalities, such as ultrasound, computed tomography (CT), and magnetic resonance imaging (MRI), not only aid in the early detection of organ involvement but also help assess the extent of disease, guide biopsy procedures, and monitor treatment response. In resource-limited settings, chest radiography and ultrasonography provide initial clues, while CT and MRI offer detailed anatomical evaluation, particularly in the CNS, skeletal system, and abdominal TB. Given these advantages, radiological profiling is essential for a comprehensive understanding of EPTB in the elderly, complementing clinical and laboratory assessments. Treatment generally follows standard anti-tubercular protocols, though extended regimens may be required for certain forms such as CNS or skeletal TB. Despite progress in TB control, the decline in EPTB incidence has been slower, and drug-resistant cases are becoming more common. Current research efforts focus on improving diagnostic tools, optimizing treatment duration based on disease site, understanding host-pathogen interactions, and addressing the impact of comorbidities on disease progression and therapeutic response.

EPTB, unlike pulmonary TB (PTB), often presents with non-classical and subtle symptoms in the elderly, making diagnosis challenging and contributing to increased disability and mortality. Older adults are particularly vulnerable due to overlapping comorbidities, polypharmacy, and age-related decline in organ function, which also heighten the risk of adverse drug reactions. These factors frequently result in delayed diagnosis, prolonged hospitalization, and poorer outcomes compared to younger patients. Studies have demonstrated markedly higher mortality in elderly individuals [2], with data from low-prevalence countries showing that nearly 80% of TB-related deaths occur in those aged 65 years and above [3,4]. Age also plays a critical role in complicating treatment, being associated with multiple medications, increased drug burden, and pre-existing health conditions [5]. Commonly affected sites in elderly patients include the spine, lymph nodes, and CNS, with thoracic spine involvement frequently observed. Advancing research on the coexistence of PTB and EPTB in this population is therefore essential to better understand epidemiological patterns and to guide the development of more effective prevention and management strategies.

Treatment challenges in this population stem from reduced organ function, multiple coexisting illnesses, and the use of several medications, which heighten the risk of adverse drug reactions. Additionally, issues such as poor treatment adherence and loss to follow-up are more prevalent among the elderly, further impacting recovery [6,7]. Despite these difficulties, early detection and personalized treatment approaches can lead to successful outcomes. However, the rising number of EPTB cases in this age group highlights the urgent need for age-specific diagnostic protocols and integrated care strategies [8].

The increasing prevalence of EPTB among the elderly highlights the urgent need for targeted research. However, investigations focusing specifically on this age group remain limited, as shown by Moya-Salazar et al. [9]. This study aims to investigate clinical patterns, diagnostic challenges, and associated risk factors in elderly patients with EPTB, addressing a critical gap in current knowledge. The findings will support the development of age-sensitive diagnostic and management strategies. The paper is structured into sections covering introduction, methodology, results, discussion, and conclusion for comprehensive analysis.

## Materials and methods

Study design

This prospective observational study was carried out over 15 months, from January 2019 to March 2020, at Rajiv Gandhi Government General Hospital, Chennai, India. A total of 120 patients aged 60 years and above, diagnosed with extrapulmonary or disseminated TB and willing to participate, were enrolled using a consecutive sampling method. Exclusion criteria included patients who declined consent and those who were lost to follow-up before the study period ended. Each participant was thoroughly briefed about the study, and informed consent was obtained. Detailed clinical histories were recorded, including presenting symptoms, duration of illness, constitutional complaints, prior TB treatment, exposure to sputum-positive cases, and the presence of comorbidities such as diabetes, hypertension, chronic kidney disease, cardiovascular and cerebrovascular disorders, HIV/AIDS, malignancies, and immunosuppressive therapy. Lifestyle factors like smoking, alcohol use, and other addictions were also documented. All patients underwent general and focused clinical examinations. Routine investigations included chest X-ray (posteroanterior (PA) view), complete blood count, random blood sugar, and serological testing for HIV, hepatitis B surface antigen (HBsAg), and hepatitis C virus (HCV). Additional tests, such as fasting and postprandial blood sugar levels, liver and renal function tests, serum electrolytes, sputum for acid-fast bacillus (AFB), ultrasonography of the neck and abdomen, spinal X-rays, and CT/MRI scans of the chest, abdomen, spine, or brain, were performed based on clinical indications.

Statistical analysis

The collected data were compiled using Microsoft Excel (Microsoft Corp., Redmond, WA, US) and analyzed using SPSS software version 14.0 (SPSS Inc., Chicago, IL, US). Descriptive statistics were used to summarize demographic and clinical characteristics, with categorical variables expressed as frequencies and percentages and continuous variables as means with standard deviations. Associations between variables were tested using appropriate statistical methods, and a p-value of <0.05 was considered statistically significant.

Governance and potential bias

Patient confidentiality, data integrity, and clinical assessments were carefully monitored throughout. As with any observational study, certain biases may have influenced the outcomes. Selection bias may have occurred due to the inclusion of only hospitalized patients diagnosed by the attending physician, potentially limiting generalizability. Recall bias is also a consideration, as self-reported histories such as prior TB treatment and lifestyle habits may be subject to inaccuracies. Observer bias could have affected clinical evaluations, given variability in physician assessments. To mitigate these risks, standardized data collection tools and consistent diagnostic criteria were employed.

Institutional review board approval

Ethical approval for the study was obtained from the Institutional Ethics Committee of Madras Medical College, Chennai-03, prior to its initiation. All participants were fully informed about the study’s purpose and procedures, and written consent was secured in accordance with ethical research standards. The study was conducted under the guidance of the Department of Geriatric Medicine, following institutional and national protocols for biomedical research.

## Results

Demographic characteristics

Of the 120 patients included in the study, 77 were male and 43 were female, giving a male-to-female ratio of approximately 1.79:1. The largest proportion of participants (47.5% (n = 57)) were aged 60-65 years, followed by 33.3% (n = 40) in the 66-70 age group, 15.8% (n = 19) between 71 and 75 years, and 3.3% (n = 4) who were older than 75. With respect to alcohol consumption, 65.8% (n = 79) were non-alcoholic, while 34.2% (n = 41) reported alcohol use. Nutritional assessment showed that 81.6% (n = 98) of patients were underweight, whereas only 18.3% (n = 22) were within the normal weight range. Additionally, 22.5% (n = 27) had a history of contact with sputum-positive TB patients, and 9.1% (n = 11) had previously received anti-TB treatment.

When examining presenting symptoms, a wide range of complaints was noted. Cough was reported in 63 patients, expectoration in 31, dyspnea in 56, and chest pain in 26. Fever was present in 47 patients, while loss of appetite (LOA) was highly prevalent in 108 patients, and loss of weight (LOW) in 93 patients. Other symptoms included local swelling or ulcers in 15 patients, abdominal distension in three patients, nausea and vomiting in four patients, and loss of consciousness (LOC) in 31 patients. Neurological symptoms were also observed, including seizures in eight patients, headache in 15, blurred vision in six, hemiparesis in seven, paraparesis in 13, and sensory disturbances in 18. Twenty-two patients reported backache, and 60 experienced a decline in activities of daily living (ADL). Altered mental status was noted in 35 patients, and one patient had a history of falls. The baseline characteristics of our patient population are reported in Table 1.

**Table 1:** Baseline characteristics DM: diabetes mellitus; SHT: systemic hypertension; CAD: coronary artery disease; CKD: chronic kidney disease; CVA: cerebrovascular accident; ADL: activities of daily living; ATT: anti-tubercular therapy

Gender category	Number of patients	Percentage
Male	77	64.2
Female	43	35.8
Age category	Number of patients	Percentage
60-65 years	57	47.50
66-70 years	40	33.33
71-75 years	19	15.83
More than 75 years	4	3.33
Alcohol consumption	Number of patients	Percentage
Non-alcoholic	79	65.8
Alcoholic	41	34.2
BMI category	Number of patients	Percentage
Normal	22	18.34
Underweight	98	81.66
Comorbidities	Yes (%)	No (%)
DM	68 (56.7)	52 (43.3)
SHT	43 (35.8)	77 (64.2)
CAD	22 (18.3)	98 (81.7)
CKD	5 (4.2)	115 (95.8)
Hypothyroidism	4 (3.3)	116 (96.7)
Hepatitis B	2 (1.7)	118 (98.3)
Immunosuppression	2 (1.7)	118 (98.3)
CVA	2 (1.7)	118 (98.3)
Past history present	Number of patients	Percentage
Contact history	27	22.5
Prior ATT	11	9.1
Symptoms	Present (%)	Absent (%)
Loss of appetite	108 (90.0)	12 (10.0)
Loss of weight	93 (77.5)	27 (22.5)
Cough	63 (52.5)	57 (47.5)
Decreased ADL	60 (50.0)	60 (50.0)
Dyspnea	56 (46.6)	64 (53.4)
Fever	47 (39.1)	73 (60.9)
Altered mental status	35 (29.1)	85 (70.9)
Loss of consciousness	31 (25.8)	89 (74.2)
Expectoration	31 (25.8)	89 (74.2)
Chest pain	26 (21.7)	94 (78.4)
Backache	22 (18.3)	98 (81.7)
Sensory disturbances	18 (15.0)	102 (85.0)
Localized swelling/ulcers	15 (12.5)	105 (87.5)
Headache	15 (12.5)	105 (87.5)
Paraparesis	13 (10.8)	107 (89.2)
Seizures	8 (6.7)	112 (93.3)
Hemiparesis	7 (5.8)	113 (94.2)
Blurred vision (new onset/increase)	6 (5.0)	114 (95.0)
Vomiting	4 (3.3)	116 (96.7)
Nausea	4 (3.3)	116 (96.7)
Abdominal distension	3 (2.5)	117 (97.5)
Fall	1 (0.8)	119 (99.2)

Clinical signs

Among the 120 patients evaluated, 77 exhibited tachycardia, 36 had elevated blood pressure, 65 presented with tachypnea, 14 showed hypoxemia, and 15 displayed signs of meningeal irritation upon admission. Routine laboratory investigations revealed a mean hemoglobin level of 10.3 g/dL, total leukocyte count averaging 8,038.75/cu mm, lymphocyte percentage at 22.39%, and an erythrocyte sedimentation rate (ESR) of 41.96 mm/hour. Biochemical parameters included a mean total bilirubin of 0.7 mg/dL, serum albumin of 3.27 g/dL, blood urea of 29.2 mg/dL, and serum creatinine of 0.8 mg/dL. Electrolyte levels showed average sodium and potassium concentrations of 129.5 and 4 mEq/L, respectively. Sputum analysis revealed that 14.2% (n = 17) of patients were AFB-positive, while 16.7% (n = 20) had *Mycobacterium tuberculosis* (MTB) detected via the cartridge-based nucleic acid amplification test (CBNAAT).

In this cohort of elderly patients with EPTB, disseminated TB was the most prevalent form (29.2% (n = 35)), followed by isolated pleural TB (25.8% (n = 31)), CNS TB (19.2% (n = 23)), skeletal TB (14.2% (n = 17)), and lymph node TB (7.5% (n = 9)). Less frequent manifestations included pericardial, abdominal, genitourinary, cutaneous, and ocular TB, each accounting for 0.8% (n = 1) of cases. Age distribution within these subtypes showed that disseminated TB was most common among patients aged 60-65 years (40% (n = 14)), while pleural TB was predominant in the same age group (51.6% (n = 16)). CNS TB cases were largely concentrated between 60 and 70 years, and lymph node TB was most frequent among those aged 60-65.

**Figure 1:**
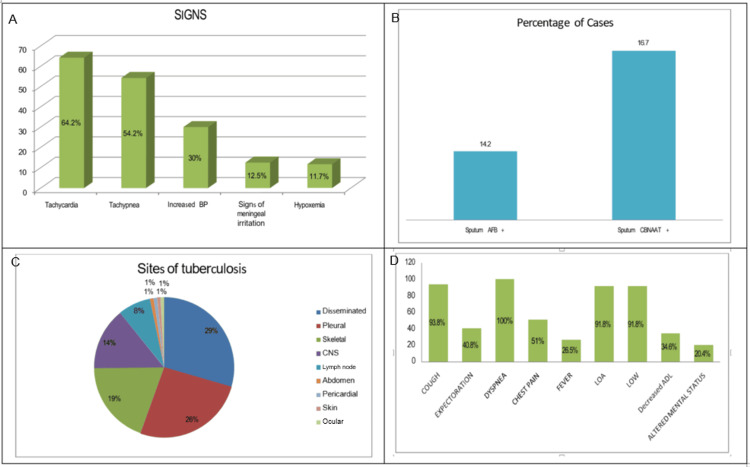
(A-D) Clinical signs, site distribution, symptomatology, and sputum analysis of extrapulmonary tuberculosis CNS: central nervous system; AFB: acid-fast bacillus; CBNAAT: cartridge-based nucleic acid amplification test; LOA: loss of appetite; LOW: loss of weight; ADL: activities of daily living; BP: blood pressure

Of the 120 patients, 49 had pleural involvement, 31 with isolated pleural TB, and 18 as part of disseminated disease, amounting to 40.8% (n = 49) of the total cohort. Among these, dyspnea was universally present, followed by cough (93.8% (n = 46)), weight loss and appetite loss (91.8% (n = 45 each)), chest pain (51% (n = 25)), expectoration (40.8% (n = 20)), fever (26.5% (n = 13)), reduced daily functioning (34.6% (n = 17)), and altered mental status (20.4% (n = 10)). Pleural fluid analysis in these cases showed mean glucose levels of 100.53 mg/dL, protein at 4.9 g/dL, lactate dehydrogenase (LDH) at 460.6 IU, adenosine deaminase (ADA) at 58.2 IU, cell count averaging 97.04 cells/mm³, and lymphocyte percentage at 58.04%. All pleural fluid samples were negative for AFB on smear, though MTB was detected in three samples. The above-discussed clinical findings are presented in Figure 1. Accordingly, Table 2 represents routine laboratory investigations, Table 3 represents the age distribution of different sites of TB, and Table 4 represents pleural involvement.

**Table 2:** Routine laboratory investigations SD: standard deviation; ESR: erythrocyte sedimentation rate

Laboratory investigation	Mean ± SD (n = 120)	Normal reference range
Hemoglobin (g/dL)	10.3 ± 1.99	12–16 (female), 13.5–17.5 (male)
Total leukocyte count (TLC) (/µL)	8,038.75 ± 2,240.17	4,000–11,000
Lymphocyte % (L%)	22.39 ± 8.7	20–40
ESR (mm/hr)	41.96 ± 12.67	<20 (male), <30 (female)
Total bilirubin (mg/dL)	0.7 ± 0.3	0.3–1.2
Albumin (g/dL)	3.27 ± 0.49	3.5–5.0
Urea (mg/dL)	29.2 ± 10	15–40
Creatinine (mg/dL)	0.8 ± 0.4	0.6–1.3
Sodium (mmol/L)	129.5 ± 5.7	135–145
Potassium (mmol/L)	4 ± 0.55	3.5–5.0

**Table 3:** Age distribution of different sites of tuberculosis CNS: central nervous system

Site of tuberculosis	Age category				p-value
	60-65 years	66-70 years	71-75 years	More than 75 years	
Disseminated	14, 40%	9, 25.7%	10, 28.6%	2, 5.7%	0.002*
Pleural	16, 51.6%	7, 22.6%	7, 22.6%	1, 3.2%	
CNS	10, 43.5%	11, 47.8%	2, 8.7%	0, 0.0%	
Skeletal	9, 52.9%	8, 47.1%	0, 0.0%	0, 0.0%	
Lymph nodes	5, 55.6%	3, 33.3%	0, 0.0%	1, 11.1%	
Pericardium	0, 0.0%	1, 100.0%	0, 0.0%	0, 0.0%	
Abdomen	1, 100.0%	0, 0.0%	0, 0.0%	0, 0.0%	
Genitourinary	0, 0.0%	1, 100.0%	0, 0.0%	0, 0.0%	
Skin	1, 100.0%	0, 0.0%	0, 0.0%	0, 0.0%	
Ocular	1, 100.0%	0, 0.0%	0, 0.0%	0, 0.0%	

**Table 4:** Pleural involvement TB: tuberculosis

Site of tuberculosis	N = 120 (no. of patients)	Percentage
Pleural involvement	49	40.8
Isolated pleural	31	25.8
Pleural TB as a part of disseminated disease	18	15.0

Radiological findings

Radiologically, a total of 27 patients underwent MRI of the brain. Among them, meningeal enhancement was observed in 17 patients (62.9%), with an equal number showing ventricular enlargement (62.9%) and periventricular edema (62.9%). Infarcts were detected in 10 patients (37.0%). Tuberculomas were also identified in 10 patients (37.0%), of which seven (25.9%) presented with a single lesion and three (11.1%) with multiple lesions. In addition, contrast-enhanced CT (CECT) of the abdomen revealed moderate ascites in two cases, massive ascites in one case, omental thickening and strands in two patients, and mesenteric lymphadenopathy in three patients.

Microbiological confirmation rates in this cohort, 14.2% positivity for AFB (n = 17) and 16.7% detection of MTB via CBNAAT (n = 20), are comparable to those found in similar studies, which often report low detection rates due to the paucibacillary nature of EPTB. This highlights the growing reliance on molecular diagnostics for accurate identification. Disseminated TB was the most prevalent form in this study, followed by pleural, CNS, and skeletal involvement. This contrasts with findings from other institutions, where lymph node TB is often more common. The higher incidence of disseminated and CNS TB here may reflect age-related immune decline and diagnostic delays in older populations.

Pleural TB was observed in 40.8% (n = 49) of cases, with symptoms such as breathlessness, cough, weight loss, and appetite loss-consistent with established clinical profiles. Pleural fluid analysis revealed elevated ADA levels, high protein content, and lymphocyte predominance, supporting standard diagnostic criteria. Although AFB smears were negative, MTB detection in select samples reinforces the value of nucleic acid amplification tests. In summary, this study supports existing evidence while offering valuable insights into the presentation and distribution of EPTB in elderly patients. It emphasizes the need for age-sensitive diagnostic approaches and greater awareness of atypical manifestations in this vulnerable group.

**Figure 2:**
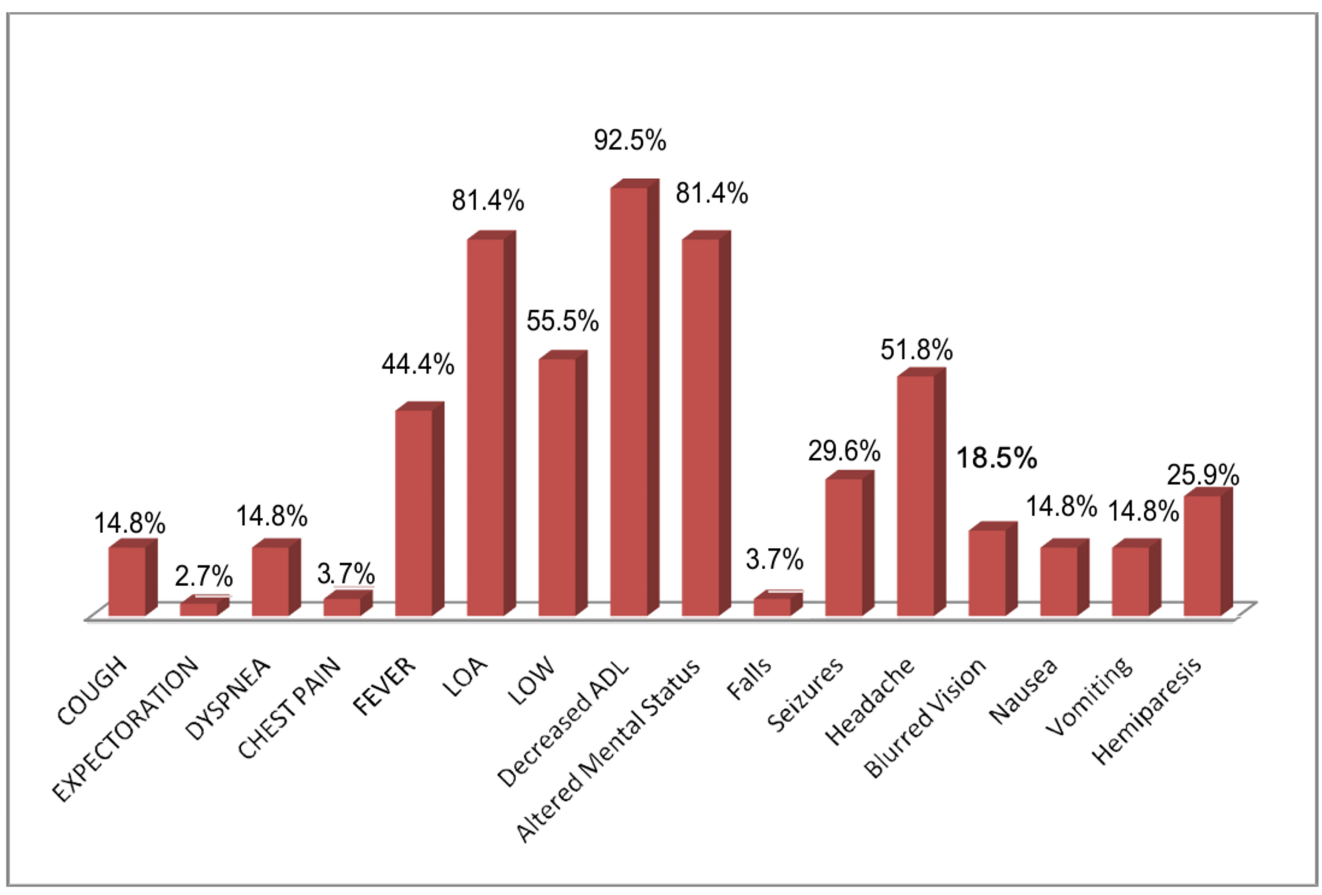
Presenting symptoms in cases with CNS involvement (%) LOW: loss of weight; LOA: loss of appetite; ADL: activities of daily living; CNS: central nervous system

**Figure 3:**
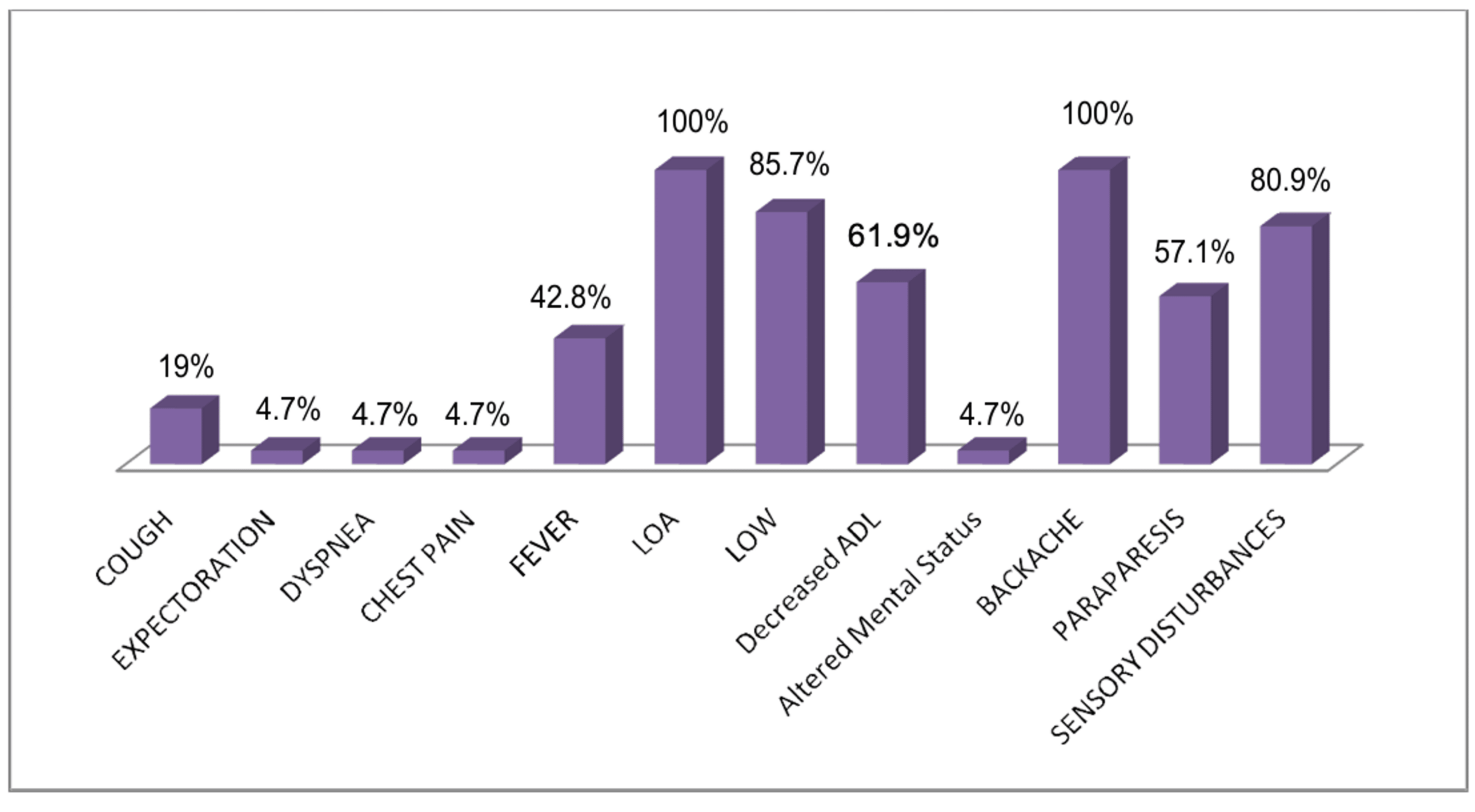
Presenting symptoms in cases with skeletal involvement (%) LOW: loss of weight; LOA: loss of appetite; ADL: activities of daily living

**Figure 4:**
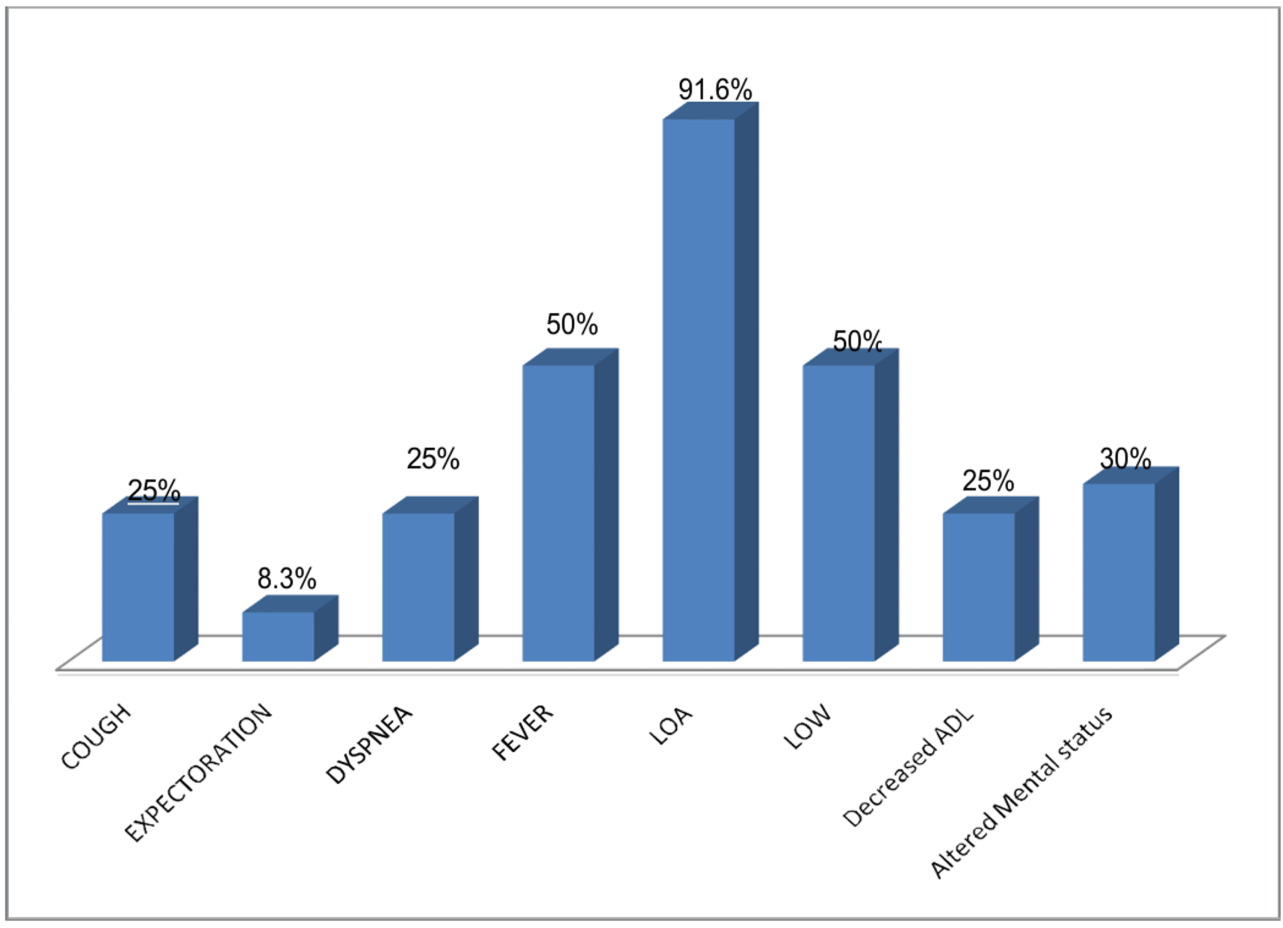
Presenting symptoms in cases with lymph node involvement (%) LOW: loss of weight; LOA: loss of appetite; ADL: activities of daily living

**Figure 5:**
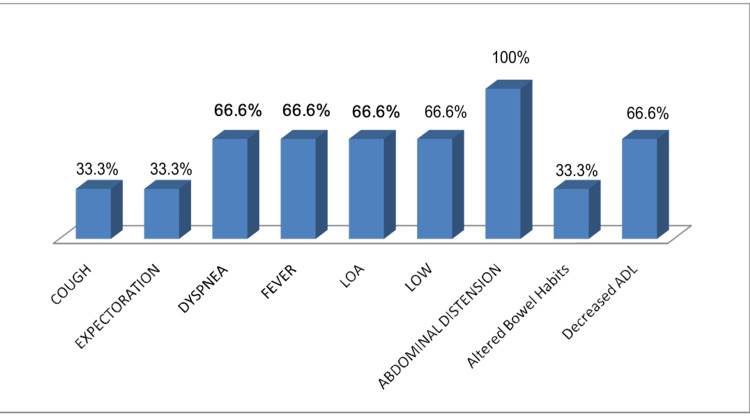
Presenting symptoms in cases with abdominal involvement (%) LOW: loss of weight; LOA: loss of appetite; ADL: activities of daily living

**Figure 6:**
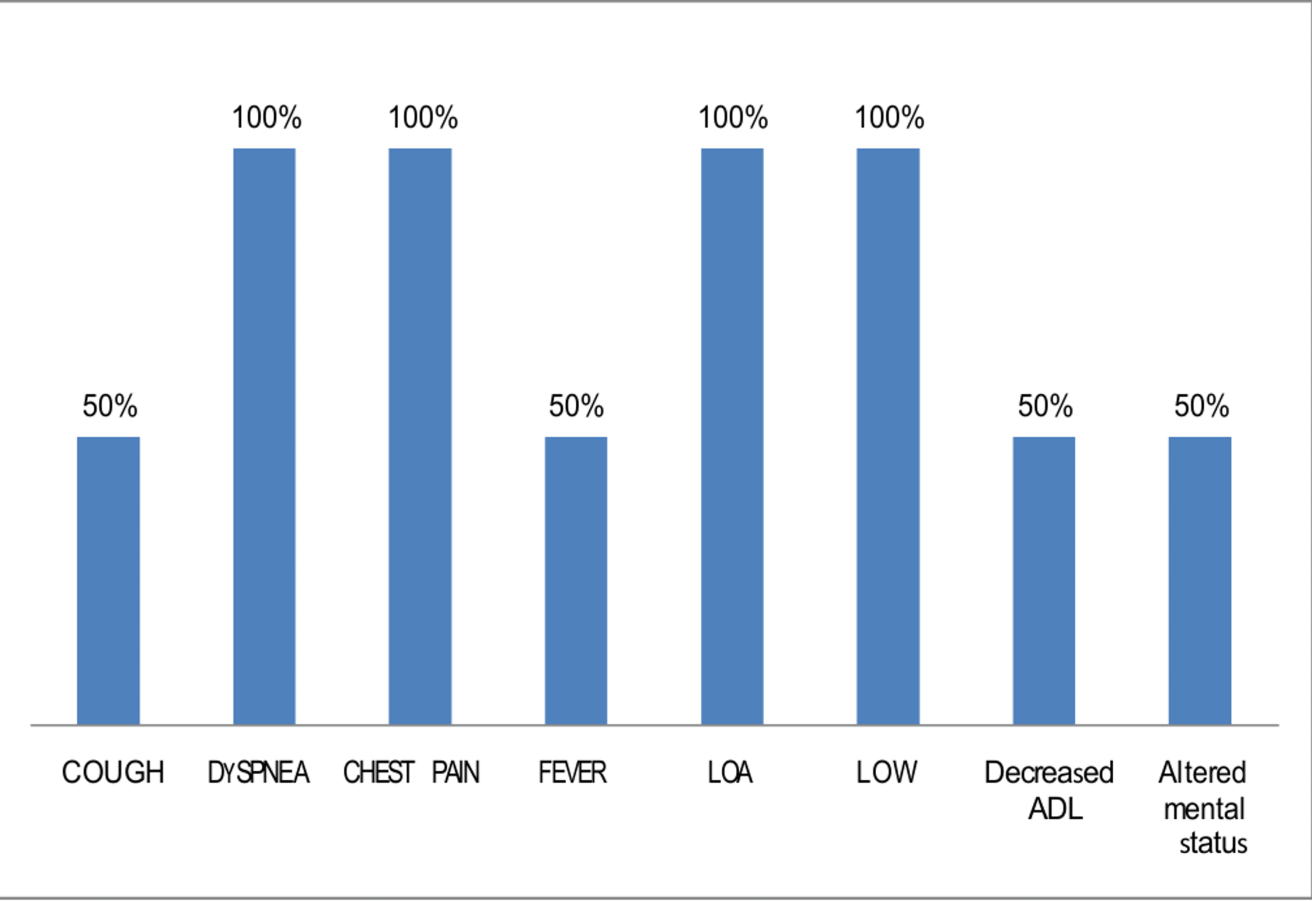
Presenting symptoms in cases with pericardial involvement (%) LOW: loss of weight; LOA: loss of appetite; ADL: activities of daily living

**Figure 7:**
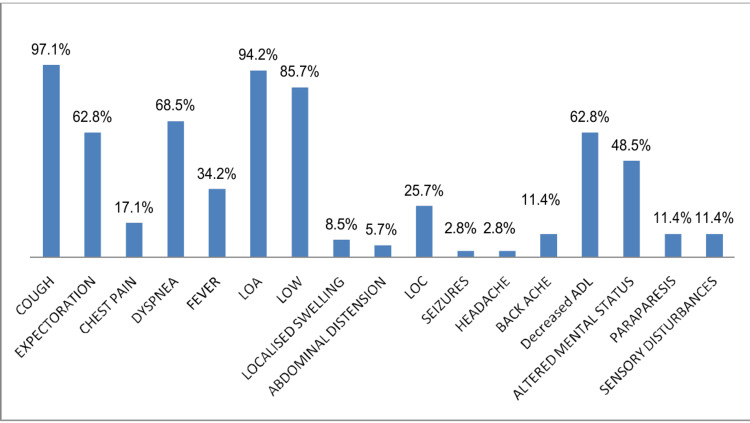
Presenting symptoms in cases with disseminated tuberculosis (%) LOW: loss of weight; LOA: loss of appetite; ADL: activities of daily living; LOC: loss of consciousness

In this study, one patient was diagnosed with cutaneous TB. The individual had previously undergone treatment for PTB 16 years ago and presented with a hyperkeratotic skin lesion. Histological analysis revealed a thickened epidermis accompanied by epithelioid granulomas and lymphocytic infiltration, findings consistent with tuberculosis verrucosa cutis. Another case involved ocular TB. The patient reported swelling, redness, and blurred vision in the left eye, along with low-grade fever and headache. Biopsy results showed granulomatous inflammation and lymphocytic infiltration, pointing toward a tuberculous origin. In Figure 2, we display the presenting symptoms in cases with CNS involvement, Figure 3 with skeletal involvement, Figure 4 with lymph node involvement, Figure 5 with abdominal involvement, Figure 6 with pericardial involvement, and Figure 7 with disseminated TB.

## Discussion

EPTB in older adults presents distinct challenges in both diagnosis and treatment, largely due to its subtle clinical manifestations, compromised immune function, and the presence of multiple coexisting illnesses [10]. This 15-month observational study at a tertiary care hospital, Chennai, involved 120 patients aged 60 and above diagnosed with EPTB. The study explored clinical symptoms, laboratory results, comorbidity profiles, and treatment histories to identify key trends and risk factors. Overall, the findings highlight the necessity for age-sensitive diagnostic strategies and holistic treatment approaches to improve prognosis in elderly individuals affected by EPTB.

This study, comprising 120 patients, revealed a male predominance with a gender ratio of 1.79:1, which is consistent with broader epidemiological patterns observed across India. For instance, a study done by Mohidem et al. [11] reported a slightly lower male-to-female ratio of 1.25:1, indicating regional variation in gender distribution among TB cases. The age profile of the participants showed that the majority were between 60 and 70 years old, aligning with national and international trends indicating a higher incidence of TB among senior age groups [12].

In terms of lifestyle factors, 34.2% (n = 41) of the patients reported alcohol consumption higher than the 15%-25% range documented in other regional studies by Lee et al. and Niu et al. [13,14], suggesting a potentially stronger link between alcohol use and TB susceptibility in this population. Nutritional assessment showed that 81.6% of patients were underweight, reinforcing the well-established association between malnutrition and increased risk of TB.

Exposure history indicated that 22.5% (n = 27) of patients had contact with individuals who were sputum-positive for TB, which aligns with WHO findings that emphasize household transmission as a significant risk factor. Additionally, 9.1% (n = 11) of patients had previously undergone anti-TB treatment, a figure slightly below the 12%-15% re-treatment rates reported in urban studies by Kapwata et al. [15], possibly reflecting better treatment adherence or fewer relapses in this cohort.

Clinically, the most common symptoms included cough, fever, weight loss, and appetite loss-hallmark features of TB that are widely reported in existing literature by Hassan et al. [16]. Notably, a substantial number of patients presented with neurological symptoms such as seizures, altered mental status, hemiparesis, and paraparesis, indicating a significant burden of EPTB. Overall, the study’s findings are in line with existing research while also offering unique insights into demographic and clinical patterns that may inform future public health strategies and clinical management approaches.

The clinical and laboratory findings in this study show strong alignment with existing research on EPTB. Common symptoms such as increased heart rate, rapid breathing, and elevated blood pressure reflect the systemic nature of EPTB and are consistent with patterns observed in tertiary care settings [17]. Though less frequent, signs of meningeal involvement point to CNS TB, echoing previous studies that highlight its diagnostic complexity. In our cohort, this challenge was particularly evident among elderly patients.

Laboratory indicators such as mild anemia, raised ESR, and low serum albumin are typical of chronic inflammatory conditions and mirror findings from high-burden TB regions. These markers emphasize the role of nutritional status and systemic inflammation in disease progression.

Microbiological confirmation rates in this cohort, 14.2% positivity for AFB (n = 17) and 16.7% detection of MTB via CBNAAT (n = 20), are comparable to other studies, which often report low yields due to the paucibacillary nature of EPTB. This highlights the importance of molecular diagnostics in improving detection.

Disseminated TB was the most prevalent form in this study, followed by pleural, CNS, and skeletal involvement. This differs from the findings of the study by Zangpo et al., where lymph node TB was more common [18]. The predominance of disseminated and CNS TB here may reflect age-related immune decline and delays in diagnosis among older adults, both of which predispose to more advanced disease at presentation.

Patients who had pleural TB presented with symptoms such as breathlessness, cough, weight loss, and reduced appetite consistent with known clinical profiles. Pleural fluid analysis revealed elevated ADA, high protein levels, and lymphocyte predominance, supporting standard diagnostic criteria. Although AFB smears were negative, MTB detection in select samples underscores the value of nucleic acid amplification tests [19]. In summary, this study reinforces existing knowledge while offering important insights into the presentation of EPTB in elderly populations. It highlights the need for age-sensitive diagnostic approaches and greater awareness of atypical manifestations.

Our study has documented two rare manifestations of EPTB. One patient presented with cutaneous TB, having previously undergone treatment for PTB 16 years earlier. The skin lesion was hyperkeratotic, and histopathological analysis revealed epidermal thickening, epithelioid granulomas, and lymphocytic infiltration findings consistent with tuberculosis verrucosa cutis. These features align with existing literature [16,20], which describes this form of cutaneous TB as uncommon and typically occurring in individuals with prior TB exposure and intact immunity [5,14].

Another case involved ocular TB. The patient experienced redness, swelling, and blurred vision in the left eye, along with low-grade fever and headache. Biopsy results showed granulomatous inflammation and lymphocytic infiltration, pointing to a tuberculous origin. This presentation corresponds with previous studies that highlight ocular TB as a rare but serious condition, often manifesting as uveitis or other inflammatory eye disorders. Literature by Nishal et al. and Bouzouita et al. [21,22] also emphasizes the diagnostic challenges due to the frequent absence of pulmonary involvement and the reliance on histopathology and molecular testing for confirmation.

Our findings highlight that the clinical presentation of EPTB in the elderly differs significantly from that in younger populations. Fever was less common, while altered sensorium and decline in ADL were more prominent, reflecting the functional vulnerability of this age group. Laboratory abnormalities such as hyponatremia, hypoalbuminemia, and anemia were frequent, with electrolyte disturbances occurring more commonly than in younger cohorts. Similar patterns have been observed in other studies, which also emphasize the need to consider age-specific variations in clinical and laboratory features when evaluating TB patients [23,24].

These observations warrant further investigation into the underlying mechanisms of electrolyte imbalance and functional decline in elderly TB patients, as they may have important implications for diagnosis, management, and prognosis. Together, these cases reflect the diverse clinical spectrum of EPTB and reinforce findings from earlier research regarding its atypical presentations and diagnostic complexity.

Limitations

This study has several limitations that should be considered when interpreting the findings. The observational design and relatively small sample size may limit both statistical power and generalizability. Being conducted in a tertiary referral hospital, the clinical spectrum and distribution of EPTB observed here may not fully reflect patterns in smaller centers or community settings. The lack of a comparative younger population restricts age-specific conclusions. Additionally, patients lost to follow-up before study completion were excluded, potentially introducing selection bias. The study did not evaluate the impact of comorbidities on morbidity and mortality, nor did it assess drug adherence, adverse effects, or long-term treatment outcomes. These limitations underscore the need for future multicenter studies with larger cohorts, inclusion of comparative age groups, and longitudinal follow-up to validate and expand upon these findings.

## Conclusions

In our observational study on EPTB among elderly individuals, disseminated TB emerged as the most prevalent form, likely due to the combined effects of immunosenescence and the presence of multiple comorbidities. Our findings add to the growing body of evidence that elderly patients with EPTB exhibit distinct clinical and laboratory patterns compared with younger populations. Prior studies have also reported variations in symptomatology and laboratory parameters between these groups, underscoring the importance of considering age-specific factors in both diagnosis and management. These insights highlight the need for further research into mechanisms underlying functional decline and metabolic disturbances in older TB patients, which may ultimately inform tailored strategies for early recognition and improved clinical outcomes. Additionally, a proportion of patients had previously received anti-tubercular therapy, highlighting the importance of improved methods for assessing treatment completion and identifying latent infections. Comorbidities were widespread, most commonly diabetes mellitus, hypertension, and a combination of both. These coexisting conditions may influence disease progression and complicate treatment outcomes, underscoring the importance of routine screening and integrated management of comorbidities in TB care for the elderly.
